# Supplementary material for: Primary Metabolic Response of Aristolochia contorta to Simulated Specialist Herbivory under Elevated CO2 Conditions
Source: Plants (Basel). 2024 May 24;13(11):1456. doi: 10.3390/plants13111456 (PMC11174525; doi:10.3390/plants13111456)
Supplement: Supplementary file 1 [file plants-13-01456-s001.zip › plants-2938216-supplementary.pdf]

plants

Supplementary Materials

Primary metabolic response of *Aristolochia contorta* to simulated specialist herbivore under elevated CO_2 conditions

Hyeon Jin Jeong [1,2], Bo Eun Nam [1,3], Se Jong Jeong [1,4], Gisuk Lee [5], Sang-Gyu Kim[6], and Jae Geun Kim [1,7,*]

*** Correspondence:** jaegkim@snu.ac.kr

(1)

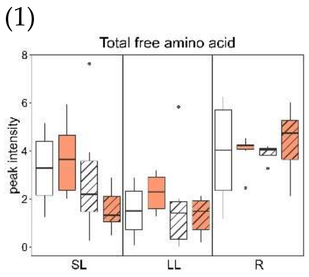

(2)

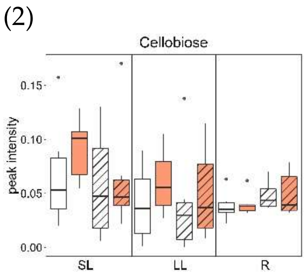

(3)

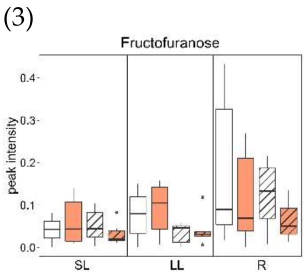

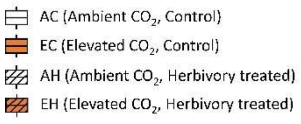

(4)

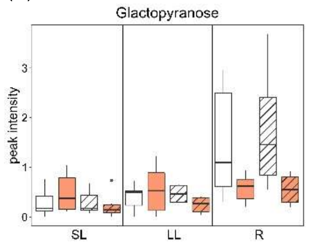

(5)

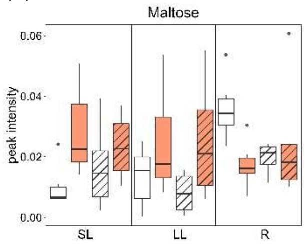

(6)

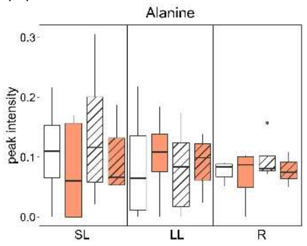

(7)

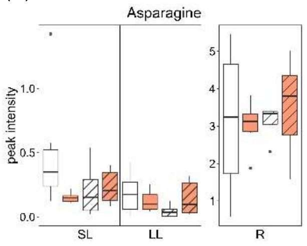

(8) (9) (10) (11)

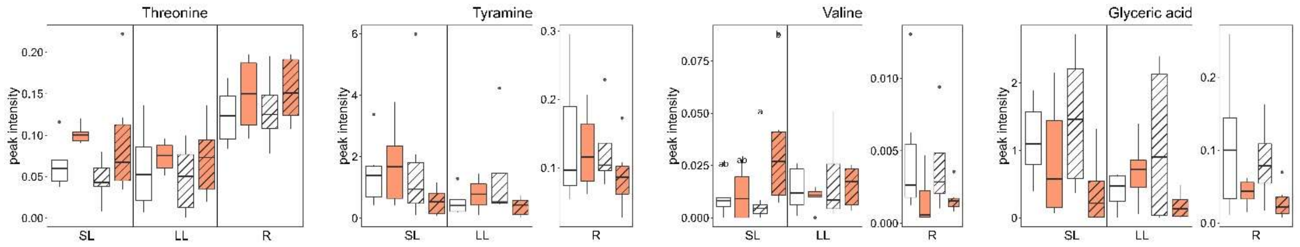

(12) (13) (14) (15)

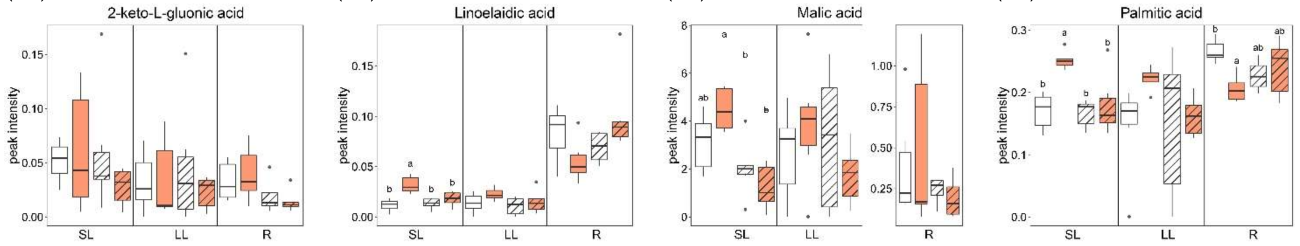

(16) (17) (18) (19)

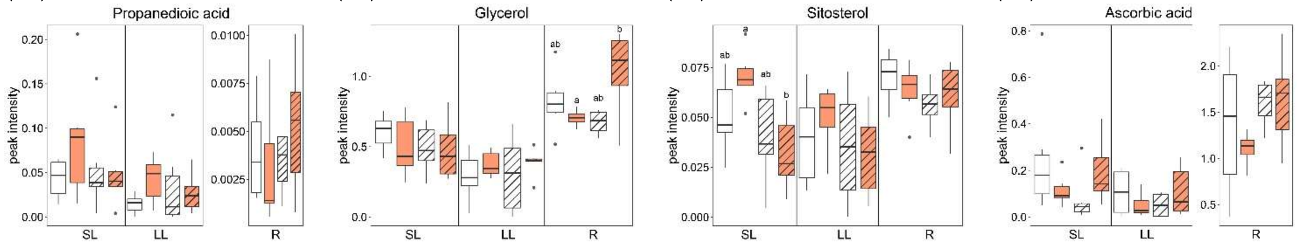

Figure S1. Concentrations of primary metabolites not documented in the manuscript, categorized by tissues types (local leaves that herbivory was treated, LL; systemic leaves, SL; roots, R) and four treatment groups (AC, EC, AH, EH). The results of Tukey's HSD test with four experimental groups are presented on the boxplot.

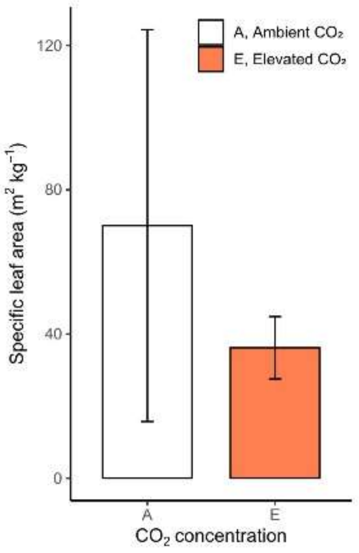

Figure S2. Specific leaf area of the plants in two CO_2 concentrations.

Table S1: Names and categories of all primary metabolites observed in *A. contorta*. Scale transformation to achieve normality for ANOVA is described.

Number	Name	Category	Scale transformation		
			Systemic leaves	Local leaves	Roots
1	Total soluble sugar		raw	raw	raw
2	Total free amino acid		raw	square root	raw
3	Allose	sugars and sugar alcohols	square root	square root	cube root
4	Cellobiose	sugars and sugar alcohols	raw	raw	reciprocal square root
5	Fructose	sugars and sugar alcohols	raw	cube root	reciprocal square root
6	Fructofuranose	sugars and sugar alcohols	square root	square root	square root
7	Glactopyranose	sugars and sugar alcohols	square root	raw	log
8	Glucose	sugars and sugar alcohols	square root	square root	raw
9	Lactose	sugars and sugar alcohols	square root	square root	raw
10	Maltose	sugars and sugar alcohols	raw	square root	square root
11	Sucrose	sugars and sugar alcohols	raw	raw	square
12	Trehalose	sugars and sugar alcohols	log	log	raw
13	Galactinol	sugars and sugar alcohols	log	square root	raw
14	Myo-inositol	sugars and sugar alcohols	raw	raw	raw
15	Ribitol	sugars and sugar alcohols	square root	raw	square root
16	Pinitol	sugars and sugar alcohols	square root	raw	raw
17	Alanine	amino acids	raw	raw	raw
18	Asparagine	amino acids	cube root	square root	raw
19	Aspartic acid	amino acids	raw	square root	cube root
20	Glutamic acid	amino acids	raw	raw	raw
21	Glycine	amino acids	reciprocal square root	reciprocal square root	raw

Number	Name	Category	Scale transformation		
			**Systemic leaves**	**Local leaves**	**Roots**
22	Threonine	amino acids	square root	raw	square root
23	Tryptophan	amino acids	log	square root	raw
24	Tyramine	amino acids	square root	cube root	square root
25	Tyrosine	amino acids	square root	square root	raw
26	Valine	amino acids	square root	square root	cube root
27	Butanedioic acid	organic acids	square root	square root	raw
28	Glyceric acid	organic acids	raw	square root	square root
29	2-Keto-L-gluonic acid	organic acids	square root	square root	square root
30	Lactic acid	organic acids	square root	square	raw
31	Linoelaidic acid	organic acids	raw	raw	square root
32	Malic acid	organic acids	raw	raw	log
33	Monopalmitin	organic acids	raw	square	reciprocal square root
34	Palmitic acid	organic acids	raw	square	raw
35	Propanedioic acid	organic acids	square root	square root	raw
36	Stearic acid	organic acids	raw	square	raw
37	Threonic acid	organic acids	square root	square root	raw
38	Glycerol	Misc	raw	raw	square root
39	Glyceryl-glycoside	Misc	square root	square root	raw
40	Octacosanol	Misc	raw	raw	square root
41	Sitosterol	Misc	raw	raw	raw
42	Tocopherol	Misc	log	square root	raw
43	Triacontanol	Misc	square root	raw	log
44	Ascorbic acid	Misc	square root	square root	raw
45	Dopamine	Misc	raw	raw	raw

Number	Name	Category	Scale transformation		
			Systemic leaves	Local leaves	Roots
46	Caffeic acid	Misc	raw	raw	cube root
47	Phosphoric acid	Misc	raw	raw	square root

Table S2: Table of the results for chlorophyll content from a two-way analysis of variance.

Source of variation	Degree of freedom	Sum of Squares	Mean of Squares	F	*p*
CO_2	1	1.30	1.30	0.06	0.80
Herbivory	1	10.50	10.55	0.52	0.48
CO_2: Herbivory	1	93.80	93.80	4.60	0.04 *
Residuals	44	897.70	20.40		

* for p-value < 0.05
